# Clinical and molecular characteristics of 69 Chinese patients with ornithine transcarbamylase deficiency

**DOI:** 10.1186/s13023-020-01606-2

**Published:** 2020-12-03

**Authors:** Deyun Lu, Feng Han, Wenjuan Qiu, Huiwen Zhang, Jun Ye, Lili Liang, Yu Wang, Wenjun Ji, Xia Zhan, Xuefan Gu, Lianshu Han

**Affiliations:** 1 Department of Pediatric Endocrinology and Genetic Metabolism, Shanghai Institute for Pediatric Research, Xinhua Hospital Affiliated To Shanghai Jiao Tong University School of Medicine, Shanghai, China; 2 Department of Neurology, Shanghai Children’s Medical Center Affiliated to Shanghai Jiao Tong University School of Medicine, National Children’s Medical Center, Shanghai, China

**Keywords:** Hyperammonemia, Urea cycle disorders, Ornithine transcarbamylase deficiency, OTC, Gene mutation

## Abstract

**Background:**

This study aimed to describe the clinical and biochemical features of Chinese patients with ornithine transcarbamylase deficiency (OTCD), and to investigate the mutation spectrum of *OTC* gene and their potential correlation with phenotype.

**Methods:**

Sixty-nine patients with OTCD were enrolled between 2004 and 2019. Clinical and laboratory data were reviewed retrospectively from medical records.

**Results:**

Fifteen cases (13 males, 2 females) presented with early onset; 53 cases (21 males, 32 females) had late onset, and one female was asymptomatic. The median onset age was 1.5 years (range 1 day–56 years). Urine orotic acid levels were increased in all patients tested, while only 47.6% of patients showed decreased serum levels of citrulline. The peak plasma ammonia levels were higher in early-onset patients than in late-onset patients (*P* < 0.01). Fifty-four different mutations of *OTC* gene were identified and 18 of them were novel. R277W (10.6%) was the most common mutation, followed by G195R (4.6%) and A209V (3.0%). By June 2019, 41 patients had survived, 24 were deceased, and 4 were lost to follow-up. Among the survivors, 13 patients had received liver transplantation at a median age of 3 years, with a one-year survival rate of 100%. The mortality of OTCD is extremely high among patients with early onset (80.0% versus 24.5% in patients with late onset).

**Conclusions:**

The evaluation of serum citrulline level is of limited value in diagnosis of OTCD, while urine orotic acid detection and genetic testing are more helpful.

## Introduction

Ornithine transcarbamylase deficiency (OTCD, MIM #300461) is one of the most common urea cycle disorders and accounts for approximately half of the inherited urea cycle disorders. The estimated prevalence is 1/14,000–1/77,000 [1]. Ornithine transcarbamylase catalyzes the formation of citrulline from ornithine and carbamyl phosphate.[2]. Thereby, OTCD leads to excess carbamoyl phosphate production, which then reacts with aspartate generating excess orotic acid. The human *OTC* gene is located on Xp21.1 with a full-length of 73 kb containing 10 exons and 9 introns [3]. Mutations of the *OTC* gene are highly heterogeneous and the recurrent sequence variants varied by ethnic background. The most common mutations are R40H and R227W in Japan [4], R129H, R40H, and G195R in Spain [5, 6], but yet unclear in China.

OTCD is an X-linked recessive genetic disorder. Therefore, almost all hemizygous males develop this disease. Approximately 20% of heterozygous females present some neurocognitive disorders due to unfavorable random X-inactivation. However, the clinical picture of carrier females is highly diverse because of the extent of X-inactivation in hepatocytes [7]. The OTCD clinical phenotypes can generally be divided into two groups, namely early-onset (onset age ≤ 30 days), and late-onset (onset age > 30 days) or asymptomatic. The major symptoms include vomiting, apastia, lethargy, convulsion, muscle hypotonia, and coma. These symptoms can be precipitated or aggravated by high protein meals, fasting, infections, trauma, surgery, or childbirth [8]. The survival rate is 70–90% among female patients, and 50–60% among male patients [9–11].

Here, we report on the clinical and biochemical features, as well as molecular characteristics of 69 unrelated Chinese OTCD patients. As such, we aimed to summarize the mutation spectrum of the *OTC* gene in Chinese patients and analyze the relationship between phenotype and genotype.

## Materials and methods

### Patients

In this study, 69 unrelated patients (34 males and 35 females) diagnosed with OTCD were recruited between 2004 and 2019. The diagnosis of OTCD was based on clinical features, specific biochemical criteria (high blood ammonia, high plasma glutamine, low plasma citrulline, and high urinary excretion of orotic acid), as well as molecular analysis. Clinical manifestations and courses, as well as biochemical data were collected and reviewed retrospectively from medical records.

### Biochemical detection

The quantification of blood amino acids was tested by tandem mass spectrometry (MS/MS; Applied Biosystems, API 4000, California, United State) on dried blood spots [12]. The levels of urinary organic acids were measured by gas chromatography -mass spectrometry (GC–MS; Shimadzu Limited, QP2010, Kyoto, Japan) [13].

### Molecular analysis of the OTC gene

After obtaining informed consent, 2 ml of peripheral blood was drawn from the patients and their parents. Samples from 66 unrelated patients were used for *OTC* molecular tests. The other 3 patients (P11-13) could not be included in the analysis as they died of acute hyperammonemic encephalopathy shortly after birth, so samples from their mothers were used for genetic test. Genomic DNA was extracted from peripheral blood leukocytes by using a blood kit (Zeesan Biotech, China). Polymerase chain reaction (PCR) was used to amplify all coding exons and exon–intron boundaries of the *OTC* gene using 9 pairs of synthetic oligonucleotide primers, and the primer sequences and annealing temperature were showed in Additional file 1: Table 1S. The PCR products were then purified and bidirectionally sequenced using the Applied Biosystems 96-capillary 3730XL system. Three patients underwent whole-exome sequencing (WES). The library was sequenced using Illumina HiSeq 4000 and generated 150 bp paired-end reads. Data analysis was performed as previously described [14]. Multiplex ligation-dependent probe amplification analysis (MLPA; SALSA MLPA P079 OTC kit, MRC-Holland, Amsterdam, The Netherlands) was performed for samples in which Sanger sequencing or WES failed to detect any disease-causing mutation [15, 16].

### Bioinformatics analysis

The variants were called novel if they were not listed in the Human Gene Mutation Database, ClinVarMiner database, and Exome Aggregation Consortium database. For the novel missense variants, potential pathogenicity was analyzed using PolyPhen-2, PROVEAN, Mutation Taster, Sorting Intolerant From Tolerant (SIFT), and Amino acid conservation was analyzed by phyloP and phastCons.

### Statistical methods

Data were expressed as the median (range, minimum and maximum) for quantitative variables and percentages for qualitative variables. The level of statistical significance was obtained using the Mann–Whitney U test for quantitative variables.

## Results

### Diagnostic mode

In this study, 15 patients underwent the newborn screening by MS/MS using blood citrulline as a biomarker. However, only 4 cases (P1, P7, P15, and P35) were identified, and 3 of them (P1, P7, and P35) with early onset phenotype showed first disease onset before the newborn screening results were available, so only one case (P15) was diagnosed through the newborn screening before the onset of symptoms. Of the other 11 patients missed by newborn screening, 9 had late onset of disease, one (P9) had severe early onset, and one (P43) was asymptomatic. Therefore, most (64/69, 92.8%) of patients were diagnosed by selective metabolic investigation after the onset of symptoms, whereas identification by newborn screening (4/69, 5.8%) or prenatal testing (1/69, 1.4%) was less frequent.

### Clinical presentations

**Fig. 1:**
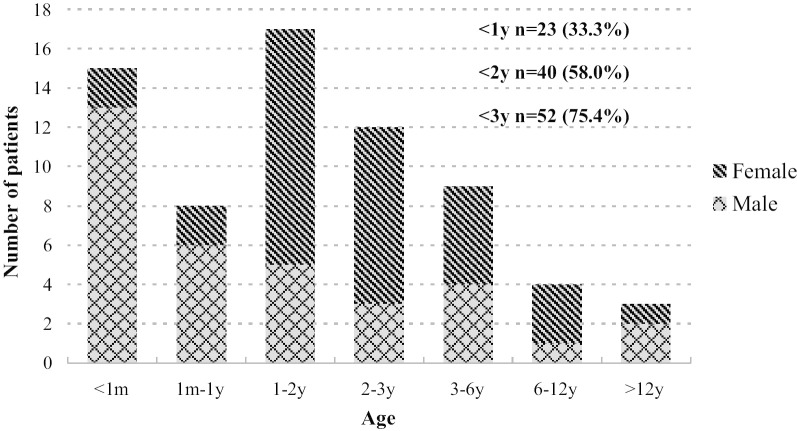
Age at onset of 68 Chinese patients with OTCD

With the exception of one asymptomatic female patient, the age at onset of 68 patients was shown in Fig. 1. Fifteen cases (21.7%), 13 males and 2 females, suffered first hyperammonemic episode during neonatal period, and the median onset age was 3 days (range 1–27 days). The remaining 53 cases (76.8%), 21 males and 32 females, had late-onset OTCD, and the median onset age was 2 years (range 3 months–56 years). Over all, three quarters of patients underwent the first hyperammonemic episode within 3 years of age, and the peak age was neonatal period in males and 1–2 years in females, respectively.

**Fig. 2:**
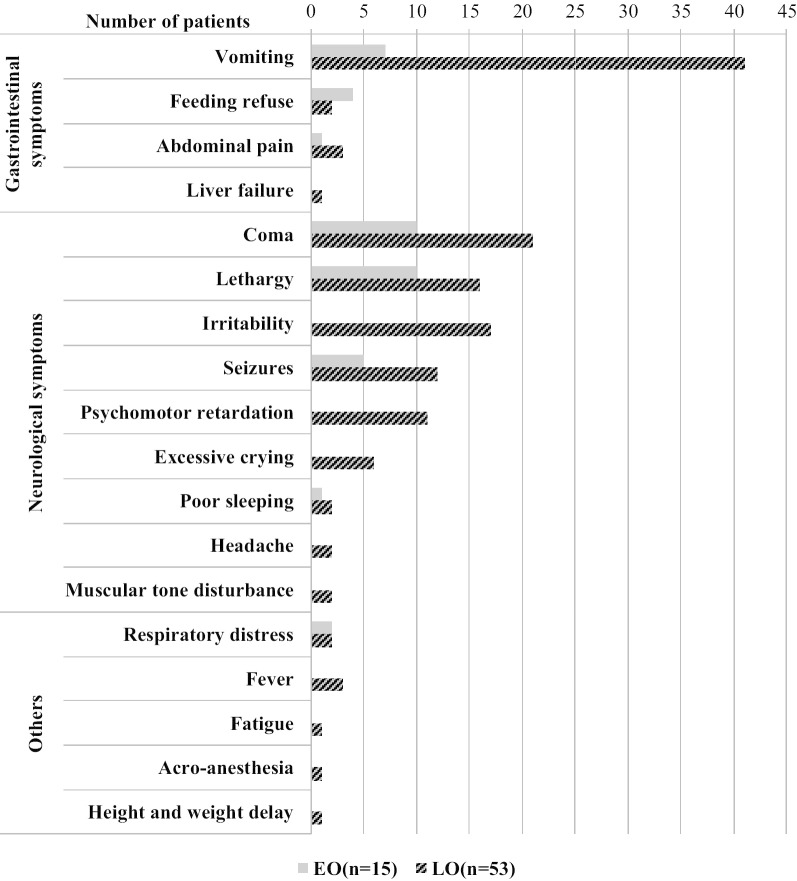
Clinical picture of OTCD patients at the moment of diagnosis

Clinical symptoms of these patients were summarized in Fig. 2. Neurological symptoms were the most common clinical manifestations in both early-onset and late-onset groups. The acute severe neurological symptoms (including coma, lethargy and seizures) occurred in 14 cases (93.3%) in the early-onset and 33 cases (62.3%) in the late-onset group, indicating clinical symptoms were more serious in early-onset group. The gastrointestinal symptoms ranked second in both groups; vomiting was the most prominent symptom.

### Biochemical results at OTCD diagnosis

The results of the biochemical studies at diagnosis were showed in Table 1. The urinary orotic acid was significantly elevated in most of tested patients (53/56) and slightly elevated in three patients (P28, P34, and P69), whereas decreased serum citrulline was found in only 47.6% (30/63) of patients. The elevated levels of blood ammonia were found in all patients tested, serum glutamine in 22.2% (12/54) of patients, and uracil in 94.4% (51/54) of patients, respectively. In addition, 73.1% (38/52) of patients had elevated plasma alanine aminotransferase (ALT), among whom 73.7% (28/38) showed severe elevation of ALT. International normalized ratio was elevated in 11 patients tested.

**Table 1:** Clinical biochemical findings at presentation in 69 patients with OTCD

Patient No	Sex	Onset age	Current/death age	Type	CIT^a^ (μmol/L)	GLN^a^ (μmol/L)	Orotic acid^a^ (mmol/molCr)	Uracil^a^ (mmol/molCr)	Peak AMON^a^ (μmol/L)	ALT^a^ (U/L)
1^b^	M	7d	8m	EO, NBS	1.71	3.25	425.42	8.60	300	32
2	M	3d	7d	EO	ND	ND	ND	ND	346	18
3	M	20d	24d	EO	ND	ND	ND	ND	ND	ND
4	M	3d	7d	EO	3.09	33.54	231.20	151.20	637	ND
5	M	9d	44d	EO	4.05	ND	ND	ND	815	ND
6	M	3d	7d	EO	2.48	80.60	267.60	53.26	700	18
7 ^b^	M	5d	3.1y	EO	3.24	15.15	22.28	8.70	50	172
8	M	1d	5d	EO	2.18	24.79	127.33	90.78	> 500	15
9 ^b^	M	3d	7d	EO,NBS	4.80	43.73	1638.32	61.91	780	ND
10	M	3d	7d	EO	4.96	ND	214.77	9.89	1015	ND
11	M	2d	6d	EO	3.25	45.78	423.71	256.54	> 500	ND
12	M	2d	2d	EO	3.40	ND	ND	ND	657	648
13	M	1d	7d	EO	ND	ND	ND	ND	1340	ND
14	M	56y	56y	LO	8.83	5.84	827.28	28.69	1152	23
15 ^b^	M	1.8y	2.3y	LO,NBS	3.07	27.20	ND	17.93	180	42
16	M	8m	2.5y	LO	ND	ND	ND	ND	300	ND
17	M	2y	*	LO	5.74	10.83	65.68	29.17	218	ND
18 ^b^	M	4m	9.7y	LO	6.83	44.39	228.79	311.67	350	28
19	M	36y	39y	LO	3.87	17.93	ND	ND	427	36
20	M	4.3y	4.3y	LO	21.74	16.37	312.35	248.40	114	244
21 ^b^	M	1.4y	4.7y	LO	5.62	9.92	413.21	143.06	351	55
22 ^b^	M	2.6y	4.4y	LO	ND	ND	ND	ND	244	ND
23 ^b^	M	1.4y	2.4y	LO	25.43	5.61	137.00	6.07	114	40
24	M	9.7y	9.9y	LO	8.58	11.23	96.00	42.90	120	69
25	M	10m	1.4y	LO	2.28	ND	ND	ND	131	327
26	M	3y	10.8y	LO	7.48	22.23	48.31	165.31	289	119
27	M	7m	2.2y	LO	1.10	14.28	543.54	130.02	392	462
28	M	1y	8.7y	LO	7.09	14.21	1.85	19.79	45	26
29	M	1.2y	8.1y	LO	3.86	15.48	455.63	133.00	187	48
30	M	8m	1.8y	LO	13.60	ND	82.82	339.11	335	1782
31	M	2.1y	3y	LO	12.77	13.53	11.60	17.53	257	116
32	M	5y	5y	LO	2.80	ND	172.80	27.80	2500	19.7
33 ^b^	M	3.4y	4y	LO	18.62	14.04	77.30	62.90	447	228.9
34	M	3 m	4.5y	LO	2.43	12.81	1.79	5.72	265	105
35 ^b^	F	1d	1m	EO,NBS	2.88	21.31	282.12	34.56	2000	ND
36	F	27d	8.8y	EO	14.83	29.73	197.00	51.80	410	2602
37	F	6y	9.6y	LO	19.24	14.22	198.45	205.32	194	509
38	F	2.5y	4.5y	LO	15.75	13.50	202.00	67.50	190	665
39	F	5.6y	5.6y	LO	12.60	11.65	189.20	50.04	500	759
40	F	2y	*	LO	14.07	18.96	141.39	35.73	ND	ND
41	F	1.5y	2y	LO	8.81	9.87	48.92	71.86	103	508
42	F	2.3y	5.1y	LO	9.70	12.78	43.50	251.00	1300	1330
43 ^b^	F	No	4m	AS,PT	6.61,	ND	224.31	ND	59	28
44	F	2.3y	3.8y	LO	11.84	4.93	43.83	131.88	120	215
45	F	2y	2.8y	LO	7.68	32.68	ND	ND	256	ND
46	F	2.4y	3.9y	LO	13.44	61.66	673.28	161.53	385	380
47	F	6m	3.8y	LO	12.68	20.81	1435.31	153.89	387	214
48	F	29y	29y	LO	8.41	18.16	38.75	51.55	421	ND
49	F	1.5y	8.4y	LO	15.90	23.87	457.51	112.32	133	ND
50 ^b^	F	2.3y	2.8y	LO	15.66	37.02	251.06	42.70	300	ND
51	F	3y	13.2y	LO	10.91	31.70	356.42	65.03	286	422
52 ^b^	F	1.5y	3.3y	LO	1.77	ND	ND	ND	350	1455.00
53	F	1.1y	3.2y	LO	4.27	ND	469.38	261.67	260	72
54	F	1.8y	9.3y	LO	5.94	15.18	204.05	83.30	216	415
55 ^b^	F	10m	2.6y	LO	ND	ND	ND	ND	315	885
56	F	3.5y	5.1y	LO	9.53	17.52	38.21	6.75	234	687
57	F	1.3y	7y	LO	8.21	26.24	227.12	441.98	189	139
58	F	1y	2.8y	LO	4.21	21.26	4.53	ND	480	97
59	F	6y	6.7y	LO	10.40	42.54	199.30	ND	170	338.2
60	F	2y	8.4y	LO	15.56	17.72	305.73	48.85	278	964
61	F	4y	9.9y	LO	5.69	23.48	19.93	338.64	> 500	839
62	F	1.1y	2.6y	LO	3.17	14.11	77.65	29.97	300	303
63	F	1.4y	7.3y	LO	8.82	49.73	691.94	559.21	197	1639
64	F	1y	7.3y	LO	3.70	5.18	55.46	156.57	383	49
65	F	9.3y	*	LO	7.44	56.51	106.88	61.87	268	ND
66 ^b^	F	1.7y	3.3y	LO	11.54	20.67	129.10	107.17	> 500	623
67	F	5.5y	5.7y	LO	7.57	19.38	308.70	78.95	231	311
68	F	2y	*	LO	6.94	29.11	213.13	164.51	256	388
69	F	1.5y	5.7y	LO	12.37	24.27	2.72	71.62	320	111

M, male; F, female; d, days; m, months; y, years; EO, early-onset; LO, late-onset; AS, asymptomatic; NBS, identified by newborn screening; PT, identified by prenatal testing; CIT, blood citrulline; GLN, blood glutamine; AMON, blood ammonia; ND, not detected

“*” donates loss to follow-up

^a^Normal reference values: CIT 7–35 umol/L; GLN 6–30 umol/L; Urine orotic acid 0–1.5 mmol/molCr; Uracil: 0–7 mmol/molCr; AMON 9–30 μmol/L; ALT 9-52U/L; severe elevation of ALT was defined as > 175 U/L in females, > 200 U/L in males

^b^The patients who underwent expanded newborn screening by tandem mass spectrometry

We also compared the biochemical data between male and female patients (Table 2). Among the patients with detected citrulline concentrations, 69.0% (20/29) of male patients and 29.4% (10/34) of female patients had decreased serum citrulline concentrations, with significantly lower levels of serum citrulline concentrations in the male group (*P* < 0.01). However, there were no significant differences in other metabolic characteristics between male and female groups (*P* > 0.05).

**Table 2:** Comparison of biochemical data between different gender groups and between different phenotypes

	CIT (μmol/L)	GLN (μmol/L)	Orotic acid (mmol/molCr)	Uracil (mmol/molCr)	AMON (μmol/L)
Reference values	7–35	6–30	0–1.5	0–7	9–30
Male (n = 34)	4.1 (29)	15.1 (23)	193.8 (24)	53.3 (25)	335 (33)
Range	1.1–25.4	3.3–80.6	1.8–1638.3	5.7–339.1	45–2500
Female (n = 34)	9.2 (34)	20.8 (31)	198.9 (32)	79.0 (29)	273 (34)
Range	1.8–19.2	4.9–61.7	2.7–1435.3	6.8–559.2	59–2000
*Z*	− 3.061	− 1.268	− 0.248	− 1.795	− 1.117
*P*	0.002	0.205	0.804	0.073	0.264
EO (n = 15)	3.3 (12)	29.7 (9)	249.4 (10)	52.5 (10)	679 (14)
Range	1.7–14.8	3.3–80.6	22.3–1638.3	8.6–256.5	50–2000
LO (n = 53)	8.4 (51)	17.7 (45)	181.0 (46)	75.4 (44)	265 (53)
Range	1.1–25.4	4.9–61.7	1.8–1435.3	5.7–559.2	45–2500
*Z*	− 3.326	− 1.938	− 1.455	− 1.336	− 3.726
*P*	0.001	0.053	0.146	0.182	0.000

Quantitative data were expressed as the median (number of patients tested) and the range (minimum–maximum)

EO, early onset patients; LO, late onset patients

In addition, the biochemical data were compared between early-onset and late-onset groups (Table 2). The peak ammonia levels in the early-onset group were much higher than those in the late-onset group (*P* < 0.01), which indicated that it may be associated with disease severity. Of all patients, 91.7% (11/12) with early-onset disease and 37.3% (19/51) with late-onset disease showed decreased citrulline concentrations. The citrulline concentration was significantly lower in patients with early-onset (*P* < 0.01). Nevertheless, blood glutamine, urine orotic acid and uracil concentration were similar between the two groups (*P* > 0.05).

### Mutation analysis of the OTC gene

We identified 54 different mutations in 63 unrelated patients including 28 (51.9%) missense mutations, 5 (9.3%) nonsense mutations, 8 (14.8%) splicing mutations, 7 (13.0%) gross deletions, 3 (5.6%) small deletions, 2 (3.7%) gross duplications, and 1(1.9%) small insertion (Table 3). In 3 patients (P67–P69), the results of WES were negative, but no MPLA was performed due to parental refusal. De novo mutations had been found in 36.2% (25/69) of the patients in this study. Mutations observed in male patients were mainly distributed in exon 6, 8, and 9, while mutations observed in females were highly dispersed. The most common mutations in our cohort were R277W (7/66, 10.6%), followed by G195R (3/66; 4.6%) and A209V (2/66; 3.0%). Of all mutations, 18 were novel: c.77+1G>C (IVS1+1G>C), c.78-2 A>G (IVS1-2A>G), c.122A>C (p.D41A), c.124_126delCTT (p.42delL), c.207-226del20, c.552insGAAC (p.S185Efs*41), c.703C>T (p.Q235X), c.704A>C (p.Q235P), c.860C>T (p.T287I), c.868-1 G>C (IVS8-1G>C), c.913C>T(p.P305S), E1-4 deletion, E1-4 duplication, E2-6 duplication, E5-8 deletion, E7-10 deletion, E9-10 deletion, and 7.8 Mb deletion of Xp11.4p21.2, which have not been previously reported in the literature or registered in the HGMD, ClinVarMiner, ExAC and gnomAD database.

**Table 3:** Molecular detective results, family history, and clinical outcomes identified in 69 patients with OTCD

NO.^a^	Type	Exon/Intron	Nucleotide change	Amino acid change	Nature of mutation	Family history	Therapy received^b^	Outcome/neurologic damage
1	EO	E4	c.386G>A	p.R129H	NA	No	LPD, drug	Alive/no
2	EO	E5	c.482A>G	p.N161S	Inherited	Yes	ND	Deceased
3	EO	E6	c.579G>A	p.W193X	Inherited	Yes	ND	Deceased
4	EO	E6	c.583G>A	p.G195R	Inherited	Yes	ND	Deceased
5	EO	E8	c.725C>T	p.T242I	Inherited	No	LPD, drug	Deceased
6	EO	E8	c.829C>T	p.R277W	De novo	No	MV, TPN, PD	Deceased
7	EO	E8	c.860C>T	p.T287>I	Inherited	Yes	Drug	Alive/no
8	EO	E9	c.904C>T	p.H302Y	Inherited	No	Drug	Deceased
9	EO	E2-6	E2-6 duplication		Inherited	No	ND	Deceased
10	EO	E7-10	E7-10 deletion		Inherited	Yes	ND	Deceased
11	EO	NA	NA	NA	NA	No	ND	Deceased
12	EO	NA	NA	NA	NA	No	ND	Deceased
13	EO	NA	NA	NA	NA	No	Drug	Deceased
14	LO	E2	c.119G>A	p.R40H	NA	Yes	Drug	Deceased
15	LO	E2	c.122A>C	p.D41A	Inherited	Yes	Drug	Alive/yes
16	LO	E4	c.386G>T	p.R129C	Inherited	Yes	LT	Alive/yes
17	LO	E6	c.562G>C	p.G188R	Inherited	Yes	Withdraw	Withdraw
18	LO	E6	c.604C>T	p.H202Y	Inherited	No	LT	Alive/yes
19	LO	E6	c.622G>A	p.A208T	Inherited	No	Drug	Alive/yes
20	LO	E8	c.794G>A	p.W265X	De novo	No	Drug	Deceased
21	LO	E8	c.829C>T	p.R277W	Inherited	No	LPD, drug	Alive/no
22	LO	E8	c.829C>T	p.R277W	Inherited	No	LT	Alive/yes
23	LO	E8	c.829C>T	P.R277W	Inherited	No	LPD, drug	Alive/no
24	LO	E8	c.829C>T	P.R277W	Inherited	Yes	Drug	Alive/yes
25	LO	E8	c.829C>T	p.R277W	Inherited	No	LPD, drug	Deceased
26	LO	E8	c.829C>T	p.R277W	Inherited	No	LPD, drug	Alive/Yes
27	LO	E9	c.913C>T	p.P305S	De novo	No	LPD, drug	Alive/yes
28	LO	E9	c.919A>G	p.K307E	Inherited	No	LPD	Alive/yes
29	LO	E9	c.931G>A	p.V311M	Inherited	No	LPD, drug	Alive/no
30	LO	E10	c.1019C>T	p.S340F	Inherited	No	LPD, drug	Alive/yes
31	LO	E9	c.929_931delAAG	p.309delE	De novo	No	Drug	Alive/no
32	LO	E1-4	E1-4 duplication		Inherited	Yes	ND	Deceased
33	LO	–	7.8 Mb deletion of Xp11.4p21.2		De novo	No	CVVH, Drug	Alive/yes
34	LO	E2	c.78-2 A>G	IVS1-2A>G	Inherited	Yes	Drug	Deceased
35	EO	I1	c.77+1G>C	IVS1+1G>C	Inherited	No	ND	Deceased
36	EO	E9-10	E9-10 deletion		De novo	No	LT	Alive/yes
37	LO	E1	c.3G>A	p.M1I	De novo	No	LPD, drug	Alive/yes
38	LO	E1	c.67C>T	p.R23X	Inherited	No	LPD, drug	Alive/no
39	LO	E2	c.140delA	p.N47Tfs*17	De novo	No	Drug	Deceased
40	LO	E2	c.174G>A	p.W58X	De novo	No	Withdraw	Withdraw
41	LO	E3	c.275G>A	p.R92Q	De novo	No	LPD, drug	Deceased
42	LO	E4	c.317G>T	p.G106V	Inherited	No	LT	Alive/no
43	AS	E5	c.421C>G	p.R141G	Inherited	Yes	Drug	Alive/no
44	LO	E5	c.422G>A	p.R141Q	Inherited	No	Drug	Deceased
45	LO	E5	c.540G>C	p.Q180H	De novo	No	LT	Alive/yes
46	LO	E6	c.552insGAAC	p.S185Efs*41	De novo	No	Drug	Deceased
47	LO	E6	c.583G>A	p.G195R	Inherited	No	LT	Alive/yes
48	LO	E6	c.583G>A	p.G195R	Inherited	No	ND	Deceased
49	LO	E6	c.626C>T	p.A209V	Inherited	No	LPD, drug	Alive/yes
50	LO	E6	c.626C>T	p.A209V	De novo	No	Drug	Alive/yes
51	LO	E7	c.704A>C	p.Q235P	Inherited	No	LT	Alive/yes
52	LO	E7	c.703C>T	p.Q235X	Inherited	Yes	CVVH, LPD, drug	Alive/yes
53	LO	E8	c.779T>C	p.L260S	Inherited	No	ND	Deceased
54	LO	E9	c.914C>G	p.P305R	De novo	No	LT	Alive/yes
55	LO	E9	c.944T>G	p.V315G	De novo	No	LT	Alive/no
56	LO	I3	c.298 + 2T>G	IVS3+2T>G	Inherited	No	LT	Alive/yes
57	LO	I3	c.298+5G>C	IVS3+5G>C	De novo	No	LPD, drug	Alive/yes
58	LO	I5	c.540+265G>A	IVS5+265G>A	De novo	No	LPD, drug	Alive/yes
59	LO	I6	c.664-1G>A	IVS6-1G>A	De novo	No	Drug	Alive/no
60	LO	I7	c.718-1G>A	IVS7-1G>A	De novo	No	Drug	Alive/yes
61	LO	I8	c.868-1G>C	IVS8-1G>C	De novo	No	Drug	Alive/yes
62	LO	E2	c.124_126delCTT	p.42delL	De novo	No	LT	Alive/no
63	LO	E2-3	c.207-226del20		De novo	No	LPD, drug	Alive/no
64	LO	E1-4	E1-4 deletion		Inherited	Yes	LPD, drug	Alive/yes
65	LO	E2-4	E2-4 deletion		De novo	No	Withdraw	Withdraw
66	LO	E5-8	E5-8 deletion		De novo	No	LPD, drug	Alive/no
67	LO	ND	ND	ND	ND	Yes	LPD, drug	Deceased
68	LO	ND	ND	ND	ND	No	Withdraw	Withdraw
69	LO	ND	ND	ND	ND	No	LT	Alive/yes

EO, early onset; LO, late onset; E, exon, I, intron; LPD, low-protien diet; MV, mechanical ventilation; TPN, total parenteral nutrition; PD, peritoneal dialysis; LT, Liver transplantation; CVVH, continuous veno venous hemodiafltration; NA, not analysed; ND, not detected

^a^Patients 1–34 are males, and patients 35–69 are females

^b^Drug: Referred to L-arginine, L-Citrulline, sodium benzoate, and sodium phenylbutyrate

The bioinformatic characteristics of the 4 novel missense variants (D41A, Q235P, T287I, and P305S) are shown in Table 4. D41 interlinks with carbamoyl phosphate domain through hydrogen bonds. The known mutant D41G has been predicted to decrease the stability of carbamoyl phosphate domain by structure based analysis [17]. Therefore, the mutant D41A may also affect the function of this domain and is predicted to be damaging. Q235 is located at the α-helix in the equatorial domain [18]. The mutant P235 may affect the folding of the equatorial domain and is predicted to be damaging by SIFT and Mutation Taster, but benign by PROVEAN and PolyPhen-2. T287 is located at the surface of the enzyme, which is close to the conserved amino acid motif Ser-Met-Gly (SMG loop) that swings towards the active site to help catalysis [19]. The mutant I287 may affect this local neighborhood structure and is predicted to be deleterious by all servers. P305 is located at the C-terminal ornithine binding domain, which contains the constant motif Leu-His-Cys-Leu-Pro. The nonpolar amino acid proline forms a rare cis-peptide bond that is found exclusively at this location, and largely determines the shape of the ornithine site. As such, P305R mutations would be highly damaging, as observed in a male patient with neonatal-onset, which has complete enzyme deficiency [18]. Consequently, the mutant residue S305, a polar and neutral amino acid, may affect the interaction of ornithine binding and is predicted to be pathogenic by all servers.

**Table 4:** Prediction of the potential pathogenic effect of novel missense variants of *OTC* gene

Mutation	Domain	PROVEAN^a^	PolyPhen-2^b^	SIFT^c^	Mutation taster^d^	Conservation
D41A	β-sheet in polar domain	Deleterious	Benign	Damaging	126	Conserved
Q235P	α-helix in equatorial domain	Neutral	Benign	Damaging	76	Conserved
T287I	Surface of the enzyme	Deleterious	Probably damaging	Damaging	89	Conserved
P305S	Ornithine binding domain	Deleterious	Probably damaging	Damaging	74	Conserved

^a^PROVEAN prediction: default threshold is − 2.5, that is variants with a score equal to or below -2.5 are considered “deleterious”, whereas variants with a score above − 2.5 are considered “neutral”

^b^PolyPhen-2 prediction: probably damaging with a score of 1, in contrast, possibly damaging with a score under 1

^c^SIFT prediction: amino acids with probabilities < 0.05 are predicted to be deleterious, whereas variants with a score above 0.05 are considered “neutral”

^d^Mutation taster prediction: scores range from 0.0 to 215. The more they score, the more deleterious protein mutations

### Clinical outcomes

**Fig. 3:**
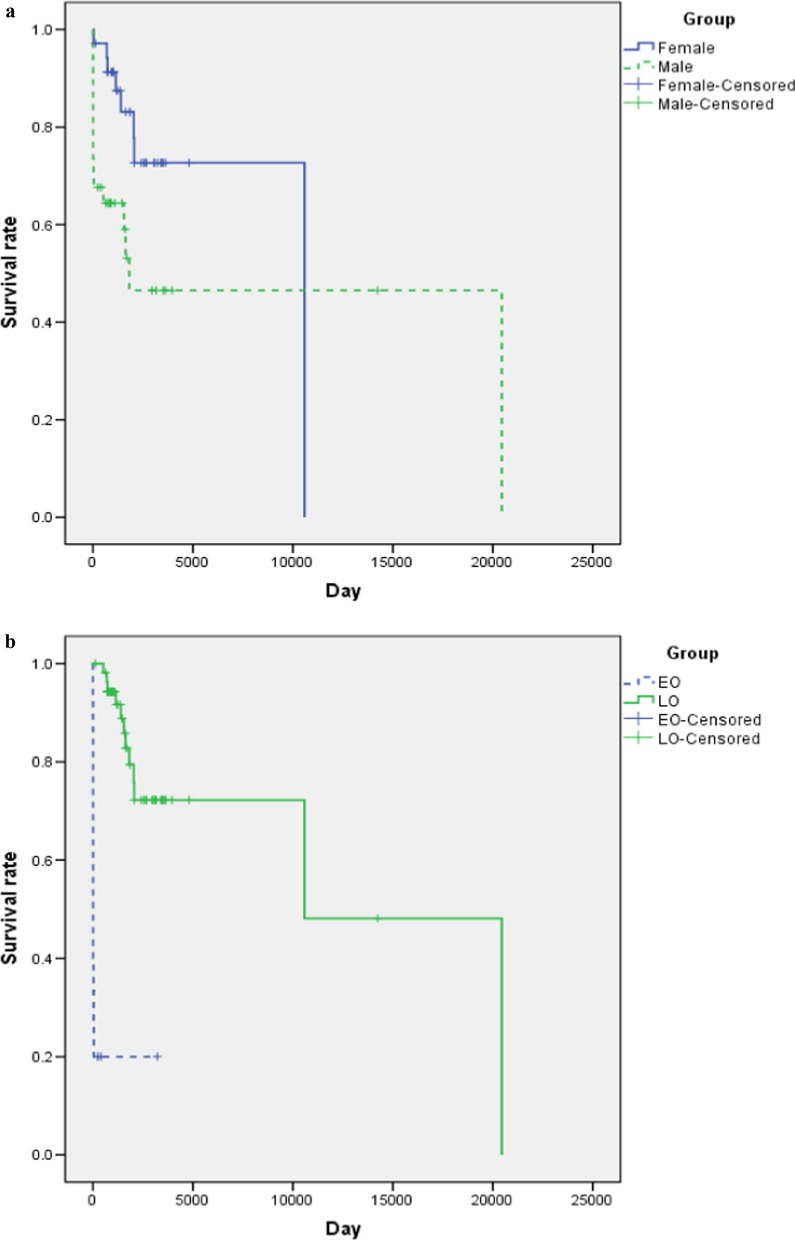
Survival rates of different groups. **a** Survival rates of male and female subjects. **b** Survival rates of early onset and late onset subjects

During 15 years of follow-up, 24 cases (16 males, 8 females) had died, 41 cases (17 males, 24 females) survived, and 4 cases (1 male, 3 females) withdrawn from this study. The mortality was 48.5% (16/33) in male patients and 25.0% (8/32) in female patients. The median age at death was 7 days (range 2 days–56 years) in males and 3.9 years (range 1 month–29 years) in females. In the early-onset group, 12 cases died following their first hyperammonemic encephalopathy at the median age of 7 days (range 2 days**-**44 days), resulting in a high mortality of 80% (12/15). In the late-onset group, except for that 4 cases lose to follow-up, 12 patients deceased at median age of 4.4 years (range 1.4 years–56 years), with a mortality of 24.5% (12/49). Estimated survival rates of these groups were shown in Fig. 3. For the 41 survivors, they survived during the initial hyperammonemic episode through nutritional support, administration of intravenous ammonia scavengers, and intensive care. Subsequent clinical progress showed neurological impairments including developmental delay, intellectual disability, learning disorder, behavior disorders, motor disorders and epilepsy, were common (65.8%, 27/41).

In this study, 13 patients (3 males, 10 females) received liver transplantation (LT). The median age at operation was 3 years (ranged from 19 months to 11.4 years). The overall median peak blood ammonia before LT was 300 µmol/L (range 216–1300) in 13 patients. Prior to transplantation, the median number of hospital admissions for hyperammonemic crises was 3 (range 1**–**12). At an average follow-up period of 1.25 years (range 0–2.7 years) following LT, the children could have a protein-unrestricted diet, with the blood ammonia being maintained in the normal range and no hyperammonemia episodes happened. Postoperative complications occurred in 2 patients, one (P18) having hepatapostema and the other (P36) having biliary fistula. Three patients (P42, P55, and P62) had good prognosis and underwent normal development. For the other 10 patients, neurological impairments were found before transplantation, and remained stable condition in 8 of them (P18, P22, P36, P45, P47, P51, P54, and P56) and got improved in 2 cases (P16, P69) after transplantation.

## Discussion

In this study, 38.2% of males and 5.7% females had early-onset presentation, 61.8% males and 91.4% of females displayed late-onset phenotype, which are similar to the characteristics reported in other studies [5]. However, we noticed that the number of males and females is almost same in our patients, which is inconsistent with the characteristic of this disease. The most possible reason is that a fraction of the male infants die undiagnosed in the neonatal period. In addition, high mortality was observed in our cohort as 80% in early-onset patients, 24.5% in the late-onset patients, respectively. Therefore, prompt diagnosis and emergency treatment are very important to reduce the high fatality rate. The diagnosis of OTCD is based on clinical symptoms, specific biochemical detection, and genetic testing. Since the clinical manifestations of this disease are non-specific, patients with unexplained vomiting and neurological symptoms need to be tested for blood ammonia levels. For patients with high blood ammonia, the levels of blood amino acids and urinary organic acid should be immediately tested. Trace amounts of serum citrulline and increased levels of urine orotic acid and uracil highly suggest the diagnosis of OTCD. However, normal citrulline levels have been detected in nearly three quarters (11/15) of our patients at neonatal screening and in approximately half of them at first onset, suggesting that identification of individuals with OTCD is unreliable by newborn screening program using citrulline as a biomarker, especially for late-onset or asymptomatic individuals. The amino acid profile by MS/MS cannot detect OTCD consistently because the monitoring of low citrulline levels lacks sensitivity and specificity [20, 21]. This is why only two UCDs (argininosuccinic synthetase and lyase deficiency) were implemented in national newborn screening panels in US and some European countries (Austria, Iceland, Portugal, Hungary and Spain) [22]. In contrast, false negatives of urinary orotic acid level was low but may occurs, as shown in some of our late-onset patients (P28, P34, P69), suggesting that multiple repeat tests are required in these highly suspicious subjects. Therefore, genetic tests should be performed for confirmed diagnosis in high-risk patient as early as possible.

The recurrent sequence variants were R277W, G195R and A209V in this study. If combining with the mutations reported by Shao et al. [23], the most frequent *OTC* mutations in Chinese population were R277W (8/90, 8.9%), R129H (4/90, 4.4%), G195R (3/90, 3.3%), A209V(2/90, 2.2%), P225L (2/90, 2.2%), and N61S (2/90, 2.2%). Herein we tried to discuss the genotype–phenotype correlations of these common mutations. R277W mutation arises in CpG dinucleotides, which recurred in discrete populations, having been reported in some male hemizygotes with asymptomatic or mild clinical manifestations [24–26]. In our study, patients with this mutant allele were all males with varied clinical symptoms. One patient (P6) experienced his first disease manifestation at 3 days old, with hyperammonemia (700 µmol/L) and cerebral edema, followed by neonatal death, even though he received mechanical ventilation, peritoneal dialysis, and parenteral nutrition. Among the other 6 male patients (P21–P26) with late-onset phenotype, one (P25) died after recurrent hyperammonemia encephalopathy at the age of 17 months; two cases (P22, P26) had intellectual disability; the remaining 3 patients (P21, P23, and P24) had good clinical outcomes through a low-protein diet and drug therapy. So, we expand the phenotype of this mutation, which can cause severe outcomes in males. The difference in phenotype with the same mutant allele may partly explain why the phenotype of patients depends not only on allelic heterogeneity but also on environmental factors, including daily protein intake [27]. The G195R mutation, affects a CpG dinucleotide [28], has been found in a male patient (P4) with early onset and 2 females (P47, P48) with late onset. P4 experienced a severe hyperammonemic episode and died a week after birth. His elder male sibling also died due to a hyperammonemia coma at 3 days following birth. P47 underwent the first hyperammonemic episode at 6 months old and experienced three instances of hyperammonemia events due to poor control of metabolic indicators via drug treatments and diet. The brain MRI showed cerebral atrophy and decreased subcortical white matter. After LT, she had good control of metabolic indexes, but little improvement in cognitive function. P48 experienced hyperammonemia during pregnancy at 29 years old, which manifested as vomiting, acro-anesthesia, limb numbness, followed by coma and rapid death. Based on the three cases described above, we suggest that this mutation could be associated with severe clinical outcomes. However, in the Spanish study, this was the third frequent mutation and had been identified in two asymptomatic female patients and one symptomatic female with late-onset phenotype [5]. As such, this indicates that the clinical outcome of this mutation may be related more to the severity at onset rather than the mutation itself. The A209V mutation occurs at a CpG site and has been reported in the Spanish population. There, one female suffered several episodes of coma from 11 to 17 months of age and remained intellectual impairment [29], while the other was a male with a late-onset phenotype, who also presented neurological damage [5]. The A209V mutation was present in two of our cases (P49, P50), both females with a late-onset phenotype. P49 was referred to us for growth delay at 18 months. Upon diagnosis of OTCD, she was prescribed a low-protein diet and treated with ammonia scavengers. However, she still experienced acute hyperammonemia crises throughout childhood. Brain MRI showed global cerebral atrophy. P50 also experienced late onset and exhibited mild intellectual impairment. The same position was also affected with pathogenic variant (A209E), which was identified in an adult female with rapidly progressive neuropsychological symptoms and coma [30]. Another variant (A209G) affects the same residue was observed in 8/92,400 (0.009%) alleles from individuals of European background, including two hemizygous individuals in gnomAD database. Although in silico analysis predicts this variant is probably damaging, the pathogenicity of A209G is unclear based on the currently available information. As such, we speculate that A209V may be associated with a late-onset phenotype in symptomatic females or males and might carry a high risk of neurological impairment. The pathogenicity of substitutions at the same position may differ greatly.

Of the 4 novel missense mutations, T287I was associated with early-onset or severe clinical manifestation. The male patient (P7) had gastrointestinal symptoms at the age of 5 days and was suspected of OTCD because of family history, with two uncles and a cousin dying of this disorder at 13 and 9 years old, respectively. Currently, he has good control of his ammonia levels and normal mental development due to early diagnosis and treatment. The other 3 novel mutations were associated with the late-onset phenotype. The D41A mutation was found in a male patient (P15) diagnosed by neonatal screening. He received conventional therapy, but metabolic characteristics were not in good control. He suffered the first onset at 1.8 years of age, and presented developmental delay and intellectual disability. His family history showed an elder male sibling died at eight months old with unclear diagnosis. The Q235P was detected in a female patient (P51). She experienced the first hyperammonemic episode at 3 years of age and suffered recurrent hyperammonemic events until accepting liver transplantation at 11.4 years of age. Poor psycho-neurodevelopment has been observed. P305S was found in a male patient (P27) with severe encephalatrophy. He had frequent epileptic seizures and cannot raise his head or sit by himself at 2.2 years old.

The gross deletions and duplications account for 16.7% of all mutations in this study. As 3 patients with undetected variants did not perform MPLA screening, the relative frequency of these mutations may be biased and thus somewhat underestimated. Therefore, MPLA analysis is necessary for all cases in which no mutation is detected by direct sequencing. Among the 9 patients with such mutations, 4 male subjects had bad clinical outcomes. Two of them (P9, P10) died from the first acute hyperammonemia encephalopathy episode in a week after birth. P32 died at 5 years old due to serious hyperammonemia (NH3, 2500 µmol/L) and had a family history of OTCD, with two uncles with similar symptoms who died during childhood. P33 survived the first hyperammonemic episode owing to urgent and continuous venovenous hemodiafiltration. However, he had severe psychomotor delays. He walks unsteadily and has poor language skills at the age of 4 years. In the 5 female cases, one (P36) displayed a neonatal phenotype and suffered recurrent hyperammonemia episodes. She received a liver transplant at 7.5 years of age and then the hyperammonemic episodes stopped. However, the impairments in cognitive function persisted, with poor performance and failing grade in grade two. Among the remaining 4 female cases (P63–P66) with late onset, 2 of them now have good control of their metabolic indexes and normal mental development; one had mild intellectual impairment and the other is lost to follow up. Therefore, we consider that gross deletion or duplication of *OTC* gene in males can cause severe clinical outcomes, while in females, the severity depends on the onset age and other factors (X-inactivation pattern or environment).

LT has emerged as a definitive treatment for the risk of metabolic decompensation in urea cycle disorders, including OTCD, with excellent post-transplant survival rates and good metabolic outcomes [31]. LT should be considered early in neonatal cases and those considered refractory to optimal medical therapy. In a study by Yu et al. [32], of the 136 patients who underwent LT for OTCD, the majority were males. However, the majority (76.9%) of our patients who underwent LT are females. This can probably be attributed to two main factors: (1) Male patients have higher mortality of 48.5% and one third of males have neonatal death following the first hyperammonemic episode. (2) Since females have higher survival rate, some of them suffered from a high frequency of hyperammonemic crises, which urged them to receive LT. It have been reported that neurologic outcomes can be improved by LT before the age of one [33], and the effects on cognition can be stabilized to premorbid function in the long term [34]. However, none of our patients received LT before 1 year of age, with the youngest pediatric LT being performed in a girl of 1 year and 7 months old. This is likely due to the shortage in the selection of the timing and indication for LT by clinicians, rather than donor shortage, economic pressures and technical reasons, because children under 1 year of age are the main (61%) recipients of pediatric LT in China after 2012, with one-year and five-year survival rates of 90.5% and 83.6%, respectively (data from China Liver Transplant Registry). In addition, the five-year survival rate and long-term neurologic outcomes of our patients remain unknown, which require longer follow-up.

In conclusion, we described clinical symptoms, biochemical features, clinical outcomes, and molecular analysis of 69 OTCD patients. There is an equal sex ratio in our patients. The mortality rate is extremely high among early-onset patients. We consider that the evaluation of serum citrulline level is of limited value in diagnosis of OTCD, while urine orotic acid detection and genetic testing are more helpful. A total of 53 different mutations have been identified and 18 of them are novel.

## Supplementary information

**Additional file 1: Table 1S.** Primers and annealing temperature for PCR amplification of OTC-specific fragments.

## Data Availability

The datasets used during and/or analyzed during the current study are available from the corresponding author on request.

## Abbreviations

F: Female
M: Male
d: Days
m: Months
y: Years
EO: Early onset
LO: Late onset
AS: Asymptomatic
CIT: Blood citrulline
GLN: Blood glutamine
AMON: Blood ammonia
ALT: Alanine aminotransferase
E: Exon
I: Intron
LPD: Low-protien diet
MV: Mechanical ventilation
TPN: Total parenteral nutrition
PD: Peritoneal dialysis
LT: Liver transplantation
CVVH: Continuous veno venous hemodiafltration
