# Supplementary material for: Clinical and molecular characteristics of 69 Chinese patients with ornithine transcarbamylase deficiency
Source: Orphanet J Rare Dis. 2020 Dec 3;15:340. doi: 10.1186/s13023-020-01606-2 (PMC7712605; doi:10.1186/s13023-020-01606-2)
Supplement: Supplementary file 1 — Additional file 1: Table 1S. Primers and annealing temperature for PCR amplification of OTC-specific fragments. [file 13023_2020_1606_MOESM1_ESM.docx]

Table 1S. Primers and annealing temperature for PCR amplification of OTC-specific fragments

| Primers | Sequence (5'→3') | Temperature (℃) |
| --- | --- | --- |
| OTC-E1F | CTGGCTAACTTGCTGTGGAG | 59 |
| OTC-E1R | AGAGTAAAGCAGAGCGTGGC | 59 |
| OTC-E2F | GGCCGGATCAACAGTAAAACAATG | 59 |
| OTC-E2R | GCCCGTATGCTCCATGATAAT | 59 |
| OTC-E3F | ACCTGGCCTAAATTCACT | 53 |
| OTC-E3R | TTCACCTTCAATCCCTCT | 53 |
| OTC-E4F | GCGGAGATGATGCCAATTCTTTGT | 57 |
| OTC-E4R | GGTTATGAGCCACTGAATGT | 57 |
| OTC-E5F | GTAAGTCGTGGGAGGTAGAACATC | 60 |
| OTC-E5R | CGGGCTAAAAGGGTTTGGGAAT | 60 |
| OTC-E6F | CCACCAAGGCACTAATACTGAG | 60 |
| OTC-E6R | GGGCCGGTAACGTAACCTAAAT | 60 |
| OTC-E7-8F | GAACATGGTGGGACCACATCTTG | 62 |
| OTC-E7-8R | GCGGGCTTGAAATTCCTTCATCCAG | 62 |
| OTC-E9F | CGCTCCCTTTGCATTGATGTCT | 59 |
| OTC-E9R | GGCATGAATGACCAAGTTGGAACC | 59 |
| OTC-E10F | TAGGTCCCTAAGCAGACTGTCGC | 62 |
| OTC-E10R | TCCTACGGTTCACCCATACCACG | 62 |
